# Aeroponic Technology for Accelerated Weathering of Extraterrestrial Regolith to Extract Plant Essential Nutrients and Generate Arable Soils

**DOI:** 10.1021/acsearthspacechem.4c00312

**Published:** 2025-02-10

**Authors:** Harrison R. Coker, Aenghus C. Denvir, Isaiah J. Robertson, Caleb E. B. Shackelford, Wen-hui Li, Chia-wei Lin, Rachel M. Watters, Donald L. Sparks, A. Peyton Smith, Julie A. Howe

**Affiliations:** † Department of Soil and Crop Sciences, Texas A&M University and Texas A&M AgriLife, 2474 TAMU, College Station, Texas 77843, United States; ‡ Department of Soil and Environmental Sciences, National Chung-Hsing University, 145 Xingda Rd., Taichung 40227, Taiwan

**Keywords:** Moon, Mars, ISRU, aeroponics, regolith, simulants, nutrients, agriculture

## Abstract

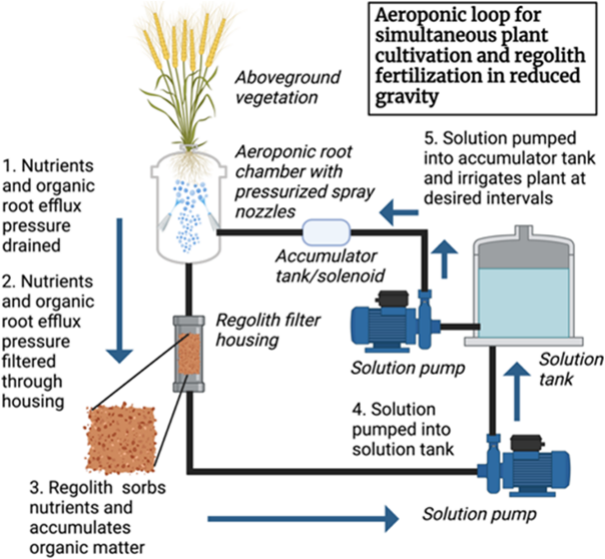

Advancements in off-world food and fiber production should seek to utilize regolith as a source of nutrients and prepare it for use as a solid plant growth substrate. Towards this goal, aeroponic biowaste streams containing both inorganic nutrients and root system efflux from plants provide an opportunity for accelerated weathering and enhancement of extraterrestrial soils. To test this hypothesis, an aeroponic system was built that contained Martian simulant (Mars Mojave Simulant-2; MMS-2), inert sand, and a no-filter control to evaluate the in-line filters for simultaneous mineral weathering and recycling of biowastes from wheat. The growth performance of wheat in aeroponics was highly productive across all treatments. After inundation with biowastes from the aeroponic system growing wheat for 40 days, MMS-2 sorbed P and K and released Al, B, Ca, Fe, Mn, Na, and S into the nutrient solution. Generated plant biowaste was mixed into MMS-2 and sand treatments, which increased the extractable Fe, K, Mg, P, and S in MMS-2. Substrate chemical properties were quantified (e.g., total C and N, total and extractable elements, pH, EC, particle size, and P species). Augmentation of MMS-2 with aeroponic biowastes followed by amendment with plant residue greatly improved wheat growth compared with the unmodified MMS-2, which resulted in plant death. This technology expands lunar/Martian base agriculture by offering a means to acquire nutrients from weathered regolith while simultaneously improving the fertility of extraterrestrial soils.

## 1 Introduction

### 1.1 In Situ Resource Utilization

Lunar and Martian colonies will need to utilize surface regolith to extract natural resources of interest in a process deemed in situ resource utilization (ISRU).^1^ From acquisition of construction materials^2^ to extraction of oxygen,^3^ ISRU offers the capability to reduce payload from Earth, which will be a defining component of lunar and Mars colonization given their considerable distance and limited launch windows. One application of ISRU is the acquisition of plant essential nutrients via direct weathering of regolith, which has been investigated in batch experiments.^4^ Regolith weathering, which is broadly the physical breakdown and chemical dissolution of constituent minerals, will be accelerated from plant growth due to root-soil interactions that plants utilize to liberate and obtain nutrients from their substrates; additionally, when combined with root-induced weathering, nutrients may rapidly dissolve from regolith on the moon and Mars given their underlying basaltic mineralogy, which is relatively easily weatherable. As nutrient acquisition from weathered regolith is therefore feasible, developing plant growth systems that weather the regolith in a passive manner presents a novel opportunity for enhancing ISRU given the abundant plant nutrients contained in the regolith.

### 1.2 Martian Regolith Mineralogy and Fertility

While crops have been reported to grow in unmodified Martian simulants,^5^ it was later discovered that the simulants used for experimentation contained nutrient artifacts that led to biased plant growth.^6^ Thus, in a later study, the addition of organic matter (OM) was found necessary to support plant life in a simulant more analogous to actual Martian regolith.^7^ Concentrations of perchlorate (ClO_4_) occurring in the regolith of Mars,^8^ and other oxidants such as hydrogen peroxide (H_2_O_2_) and clay/metal oxides,^9^ are present in toxic levels for plants and may limit the bioavailability of various metals.^10^ It has been concluded that unmodified Martian regolith is theoretically incapable of supporting plants, necessitating the use of specially selected bedrock materials that contain abundant phyllosilicates and carbonates, fertilizers of nitrogen, copper, zinc, and boron, and controlling toxic abundances of perchlorate.^6,11^ Given nutrient inputs, many Martian surface minerals can hold exchangeable nutrients essential for fertility, including plagioclase, pyroxenes, olivine, K-feldspar, Fe-smectite, mica, and chlorite. Other minerals include sulfates,^12^ carbonates,^13^ trace nitrates,^14^ oxides/oxyhydroxides, with high levels of Cl^–^ and Ca^2+^, which are mostly formed under the oxidizing conditions of Mars’ current atmosphere.^15^

### 1.3 Aeroponic-Associated Organic Wastes

Aeroponics is a soilless plant cultivation practice that supplies pressurized nutrient solution to root systems within a closed atmospheric environment without the use of aggregate medium or continuously aerated solution.^16^ Plant productivity in aeroponics has been indicated to outcompete hydroponic and soil cultivation methods^17^ likely because water, oxygen, and nutrients can be controlled to optimize growth.^18^ Aeroponics has received attention for its versatility and success in low-gravity environments, but the organic “waste” stream associated with aeroponics is not typically assessed or recycled. Rhizodeposition, the total efflux from the root system into the soil environment, occurs in both soil and soilless cultivation approaches. As root systems are rinsed in aeroponics, rhizodeposition products are suspended within the root rinse (rinsate) and can be analyzed for their metabolomic and elemental composition.^19,20^ Aeroponic systems could be designed to recirculate the solution through a filter containing extraplanetary surface minerals that would filter organic wastes otherwise leading to abrasion of pumps, clogging of spray nozzles, and irritation of mechanical components. This would also accelerate the accumulation of the OM in the regolith. The microbiome of these mineral filters may serve as a structural niche to maintain microbial populations that evolve throughout the plant’s life cycle,^21^ likely since the molecular composition of root exudates changes as plants mature^22^ and weathering of minerals may also affect microbial habitat. It has been incorrectly stated that aeroponics are void of microorganisms and are thus disadvantaged compared with soil-based growth media for filtration of bioregenerative life support systems that utilize microbial communities to degrade organic wastes.^23,24^ It is even clear that aquaponic microbiomes can be manipulated with plant growth-promoting microorganisms (PGPM) in the absence of mineral filters.^25^

### 1.4 Study Overview

As current experiments to utilize regolith for nutrient acquisition directly in plant growth systems are needed to diversify agro-agricultural operations, the goal of the study was to test the feasibility of a new plant cultivation strategy. The authors are not aware of any other studies having a similar design. The objectives were to (1) reduce nutrient payload from earth and (2) develop arable soils to support soil-based agriculture; an aeroponic system that fertilizes Martian regolith was developed and tested. Through continual inundation of organically rich rhizodeposition products and the full suite of plant essential nutrients, it is hypothesized that elements of interest can be extracted from a Martian regolith simulant while dually conferring fertility from organic wastes to support the growth of soil-based cultivation.

## 2 Materials and Methods

### 2.1 Experimental Overview

There were two phases of experimentation: (1) aeroponics were used to grow wheat while the system was filtered with either Martian simulant, sand, or a no-filter control, and (2) modified simulant was removed from the aeroponic system and used as a solid growth substrate for wheat. A Martian regolith simulant (MMS-2; “Mars Mojave Simulant”) was purchased from The Martian Garden (themartiangarden.com; Austin, TX). Perchlorate salts were not included in the simulant due to health concerns. Coarse sand was purchased from a local vendor and included as an inert control to compare with MMS-2. Aeroponic modification to filter materials occurred in a temperature modulated greenhouse (25–35 °C) from November 2022 to January 2023, amending material from January to March 2023, and growth trials of the plants in growth chambers from Apr to Dec 2023. Spring wheat (*Triticum aestivum* cv. TAMsp801) was selected as a model crop; hard red spring wheat contains the highest protein content of the wheat varieties and is typically cultivated for bread flour. Both experimental phases used pots arranged in a randomized complete block design (RCBD).

### 2.2 Augmenting Regolith with Aeroponics

**Figure 1:**
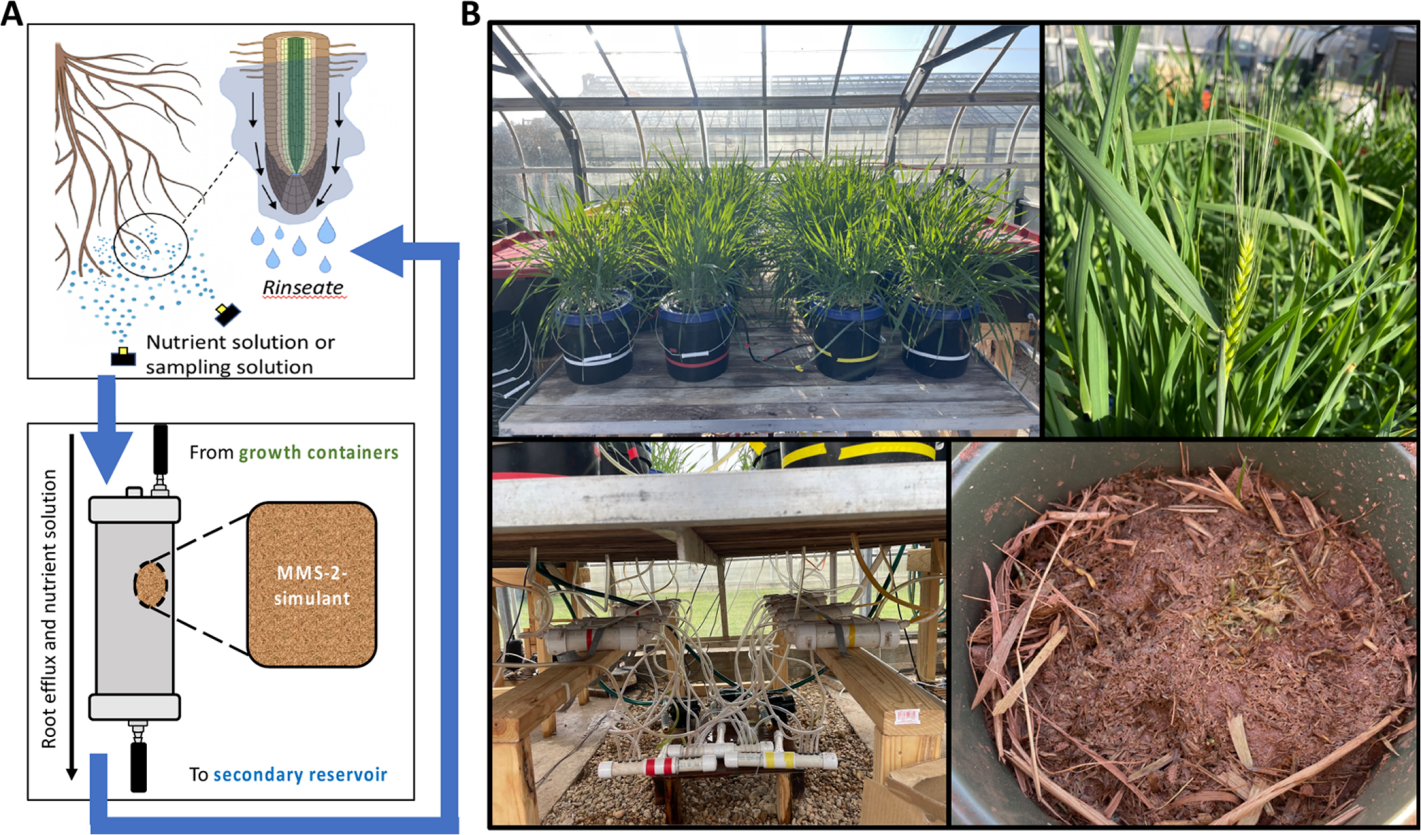
(A) Overview of the described aeroponic plant culture system equipped with a misting system that sprayed the roots. The root runoff (i.e., rinsate) containing root system efflux drained into the filter housing of the treatments containing either Martian regolith simulant, sand, or no filter. (B) Pictures of plants, aeroponic system, and residue-amended simulant during the greenhouse trial. Images captured by Harrison R. Coker.

An aeroponic system was fabricated (Figure 1) as a modified version of a previous aeroponic design.^26^ The modifications include components to make the system “closed-loop”, which includes adding a filter housing and a secondary pump to return the filtered solution back to the primary reservoirs. There were 3 treatments, each treatment having a different substrate in the aeroponic filter housing (MMS-2, sand, and a no-filter control). A 25% strength Hoagland’s solution^27^ was used to supply plant essential nutrients to plants. The entire system was drained 3 times, and the solution was replaced throughout the experiment to replenish nutrients. The pH of the solution was adjusted to pH 6.5 by using 2 M hydrochloric acid (HCl) and 2 M sodium hydroxide (NaOH). There were six wheat plants per pot. Each of the three treatments contained 8 pots that drained into their own filter housing for a total of 24 experimental units.

As plant roots were misted in aeroponics, root runoff was drained through 9.53 mm tubing into a poly vinyl chloride (PVC) filter housing that was 6.35 cm wide × 76.2 cm long, having 0.635 cm threaded push-to-connect inlets and outlets. Each pot had its own filter housing, except for control treatment, which was not filtered and freely recirculated in the system. The PVC filters contained 600 g of an MMS-2 dry simulant or terrestrial sand. After being filtered, the solution was drained into a PVC manifold through 9.53 mm rubber sealing gaskets connected to an 186.5 W (0.25 hp) water pump where the solution was pumped back into the primary reservoirs. Each treatment used an independent aeroponic system to prevent contamination. Once plants reached the flowering stage, phase one was terminated, and 20 g samples of the aeroponically altered MMS-2 and sand were taken from each filter and stored at 4 °C for subsequent analysis. Plant tissues were harvested, dried for 1 week at ambient temperature, and weighed. Plant biomass was homogenized and then sampled (10 g) for analysis. The aeroponically modified substrates were considered “augmented” for further discussion.

The dried and sieved plant biomass from each pot was directly incorporated into the filter material. To maintain organic decomposition, DI water was added to field capacity every other day until biological activity was halted. The then biomass-amended MMS-2 and sand were sampled (50 g) for analysis. The aeroponically modified and then biomass-incorporated substrates were considered “amended” for further discussion.

#### 2.2.1 Instrumentation and Analysis

To best understand the effect of aeroponic augmentation and subsequent addition of plant biomass, there were three time points for analysis of filter materials: before aeroponic filtration (unmodified), after aeroponic augmentation (augmented), and following residue addition into the augmented or amended material (amended). Total carbon (TC) and total nitrogen (TN) of filter materials and plants were measured by using flash combustion (Vario El Cube, Elementar). Permanganate oxidizable carbon (POxC) was quantified using the KMnO_4_ spectrophotometric procedure.^28^ Inorganic C was quantified by pressure calcimetry.^29^ Nitrate (NO_3_^–^) was quantified using the VCl_3_ spectrophotometric method^30^ and ammonium (NH_4_^+^) using the salicylate-nitroprusside spectrophotometric method.^31^ Extractable elements were quantified using Mehlich-III extraction^32^ and total nutrients using concentrated nitric acid with microwave digestion (EPA method 3051A) in inductively coupled plasma–optical emission spectroscopy (ICP-OES; Thermo Scientific, iCAP 7400 Duo). Total and extractable elements were obtained by the digested and extracted solutions, and a mass balance was calculated using the known amount of regolith (600 g) in each filter. To determine cation exchange capacity (CEC) the National Resources Conservation Service (NRCS) method, CEC-8.2 was used. Briefly, 2.5 g of soil was saturated with 60 mL of pH 8.2 sodium acetate over 2 h, shaken, rinsed with ethanol two times to displace excess Na, and extracted with pH 7.0 ammonium acetate. The Na concentration in the extracts was measured on flame atomic absorption spectroscopy (AAS). The pH and EC of filter materials were measured in a 1:5 soil:deionized water solution using benchtop probes.

X-ray diffraction (XRD) patterns were collected on a Bruker D8 ADVANCE diffractometer (Bruker AXS GmbH, Karlsruhe, Germany) equipped with a Cu X-ray tube operated at 40 kV and 40 mA. XRD patterns were recorded with a 2θ range between 2 and 70° with a 0.05° step size and a dwell time of 3 s. To filter out the strong Fe fluorescence from the iron oxide minerals, an energy-dispersive detector (Sol-X) was used. Mineral identification was performed with the ICDD PDF-2 database in the Bruker EVA program. Material was dried at 35 °C for 4 days, milled to powder, and formed into a pellet for XRF and XRD analysis.

Phosphorus speciation for solid samples was determined using P K-edge absorption near edge structure (XANES) spectroscopy performed at TLS beamline 16A at the National Synchrotron Radiation Research Center in Taiwan. Samples were prepared as pellets to mount in acrylic holders and covered with a polypropylene X-ray film. The synchrotron radiation was calibrated to 2222.3 eV based on the first inflection point in the L3-edge derivative spectra from a Zr foil. Spectra were acquired in fluorescence mode at photon energies from −50 to +125 eV relative to the energy of the P K-edge at 2151 eV with a step size of 0.2 eV across the near edge region (−5 to +10 eV). Multiple XANES scans were aligned, merged, baseline corrected, and normalized by using the Athena program. The phosphorus speciation was determined via linear combination fitting (LCF) for XANES data from −20 to +30 eV. The reference spectra included organic-P (phytic acid), Fe–P (phosphate adsorbed on ferrihydrite), Al–P (phosphate adsorbed on boehmite), and Ca–P (phosphate adsorbed on apatite).

#### 2.2.2 Plant Materials

Spring wheat was vernalized for 4 weeks and then germinated on filter paper with DI water. Plants were transferred to hydroponics in quarter-strength Hoagland’s solution 3 days after germination. Plants were then transferred to aeroponics 14 days after germination (once plants supported 3–4 true leaves). Plant heights were recorded three times using a meter stick and measuring the vertical axis of each plant. Whole plant dry biomass was measured after drying the plants at 30 °C for 2 days. Dried plants were shredded by using a plant grinder and sieved to pass 1 mm for analysis.

### 2.3 Plant Growth in Aeroponically Altered Regolith

Spring wheat seeds were germinated directly in the solid substrates. The amended MMS-2 and sand were compared with unmodified substrates and potting media. To obtain enough material for subsequent plant growth in the regolith, the amended substrates were composited into three pots (1.6 kg) to grow spring wheat. Wheat seeds were germinated on filter paper using DI water, and 5 seeds were transplanted to substrates after 3 days. The seedlings were reduced to 3 plants per pot after 7 days of growth in the substrate. Irrigation was provided every other day with DI water. Plant performance was monitored by measuring the canopy heights. There were 3 replicates per treatment.

### 2.4 Statistical Analysis

All data were processed in R Studio (version 4.4.1) and graphs made in *ggplot*.^33^ For canopy height in the aeroponic system and growth trial, one-way analysis of variance (ANOVA) was used with filter material as the fixed effect. Individual elemental concentrations of the augmented and residue-amended treatments were compared to the unmodified substrate using student’s *t*-test after checking assumptions of normality. Significance thresholds were set at α = 0.05.

## 3 Results

### 3.1 Plant Performance in Aeroponics

**Figure 2:**
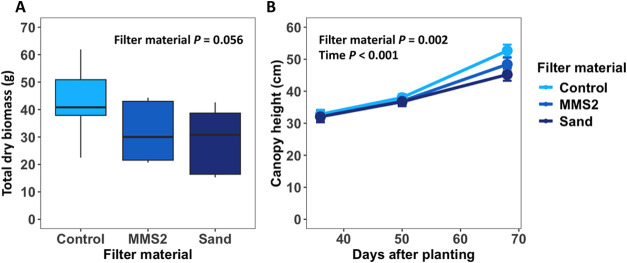
Plant productivity in the aeroponic system measured as the (A) total dry biomass and (B) canopy height. Canopy height presented as mean ± standard deviation (SD).

Aeroponic cultivation of wheat was highly productive across all treatments and advanced into grain-filling physiology (Feekes stage 11). Using MMS-2 and sand as an aeroponic filter had a marginal effect (*P* = 0.057) of reducing plant dry biomass (Figure 2A), but MMS-2 and sand were not significant from each other. As for canopy height, there was an effect of time (*P* < 0.001) indicating plants maintained growth throughout the experiment, and an effect of filter material (*P* = 0.002) as control treatment had the tallest plants while MMS-2 and sand treatment did not differ from one another (Figure 2B) by the end of the experiment. Harvest metrics were not able to be collected due to electricity loss to the aeroponic system at the greenhouse, which led to premature death of plants just prior to harvest. There was a potential outage of 12 h that ultimately led to ultimate plant senescence. However, all treatments rapidly proceeded to produce a harvestable yield with no differences in emerged heads between treatments; at the time of power loss, all pots had 3–5 grain-filling heads.

### 3.2 Carbon and Nitrogen Added to Aeroponic Modified Substrate

Plant tissue C and N did not differ among aeroponic filter treatments, and because total dry biomass did not differ, biomass from aeroponic growth was incorporated into aeroponically modified substrates without adjustments to normalize C or N (Table 1). A total of 254 g of dry biomass was incorporated into aeroponically modified MMS-2 and 229 g into aeroponically modified sand. The added biomass delivered 98.71 g C and 9.06 g N to the Martian simulant, and 89.92 g C and 7.02 g N to sand. Most likely due to microbial activity utilizing the incorporated plant material for metabolic processes, approximately 80% of C and 79% of N were lost as gases during the amendment process in MMS-2, while the sand effectively lost all added C (99%) but retained a relatively larger amount of N (66%). The evolution of C and N gases indicates net losses to the substrate after biomass incorporation, which provides insights to the sequestration potential of each material (i.e., capacity of each substrate to retain added C and N).

**Table 1:** Plant Biomass, Carbon, and Nitrogen Composition from Aeroponic Culture and Subsequent Remaining Composition following Decomposition in the Aeroponically Augmented Substrate^a^

material	biomass	element	element in plant material	added to substrate	remaining after decomposition	CO_2_ gas evolved	
	g		%	g	g	g	%
control	342	C	37.90 ± 1.17				
		N	3.55 ± 0.25				
		C:N	10.70 ± 0.93				
MMS-2	254	C	38.80 ± 0.70	98.71	20	78.7	80
		N	3.56 ± 0.19	9.06	1.92	7.14	79
		C:N	10.90 ± 0.67				
sand	229	C	39.30 ± 0.68	89.92	1.24	88.86	99
		N	3.07 ± 0.26	7.02	2.4	4.62	66
		C:N	12.9 ± 1.16				

a Because the control group did not have a filtration substrate, biomass was not used in the subsequent experiment.

The process of aeroponic augmentation did not significantly affect any of the measured C and N response variables (Table 2). However, the amended treatments led to increased total C, total N, and POxC, while the C/N ratio increased for MMS-2 and decreased for sand. No increases in NO_3_–N or NH_4_–N (not detected) in MMS-2 are evidence of an accumulation of organic N, whereas sand did have increases in NO_3_–N.

**Table 2:** Carbon and Nitrogen Characteristics of Unmodified Substrate, Augmented Aeroponically with Root Rinsate, or Amended with Residues after Augmentation^a^

material	measurement	unmodified	augmented	*P*	amended	*P*
		g kg^–1^	g kg^–1^		g kg^–1^	
MMS-2	total C	5.41 ± 0.47	5.21 ± 0.39		12.00 ± 0.85	**
	inorganic C	5.33 ± 0.26	4.84 ± 0.18		5.08 ± 0.07	
	POxC	0.07 ± 0.01	0.08 ± 0.01		0.46 ± 0.01	***
	total N	0.97 ± 0.10	0.94 ± 0.07		1.39 ± 0.06	**
	NO_3_–N	0.10 ± 0.01	0.10 ± 0.03		0.12 ± 0.01	
	C:N	5.47 ± 0.87	5.66 ± 0.60		8.66 ± 0.48	**
	total C	17.15 ± 1.71	16.86 ± 1.02		19.78 ± 1.29	*
Sand	inorganic C	18.24 ± 0.37	17.47 ± 0.53		17.82 ± 0.28	
	POxC	0.10 ± 0.01	0.08 ± 0.01		0.34 ± 0.01	***
	total N	0.66 ± 0.23	0.83 ± 0.17		1.18 ± 0.12	**
	NO_3_–N	<0.01	0.04 ± 0.02	***	0.19 ± 0.04	***
	C:N	24.96 ± 6.38	19.04 ± 1.34		16.91 ± 1.74	*

a Statistical tests (*t*-tests) were made against the unmodified material. Asterisks indicate significance at the 0.05 (*), 0.01 (**), and 0.001 (***) levels.

### 3.3 Chemical Modification of Filter Material

The pH of MMS-2 acidified as an effect of aeroponic augmentation, whereas sand became more alkaline; however, both substrates became more alkaline following residue decomposition (Table 3). Aeroponic augmentation reduced the EC of MMS-2 seven times and sand two times, which likely reflects differences in initial EC in unmodified materials. Residue amendment further raised the EC of both treatments. The CEC of both treatments was reduced during aeroponic augmentation but increased upon residue amendment, similar to pH.

**Table 3:** Substrate pH, Electrical Conductivity (EC), and Cation Exchange Capacity (CEC) of Unmodified Substrate, Augmented Aeroponically with Root Rinsate or Amended with Residues after Augmentation^a^

material	measurement	unmodified	augmented	*P*	amended	*P*
MMS-2	pH	10.0 ± 0.1	9.1 ± 0.1	***	9.6 ± 0.1	***
	EC (mS cm^–1^)	2.8 ± 0.5	0.4 ± 0.1	***	1.7 ± 0.8	**
	CEC (meq 100 g^–1^)	13.1 ± 1.1	11.4 ± 1.5	*	13.7 ± 2.0	
sand	pH	8.8 ± 0.1	9.4 ± 0.1	***	10.4 ± 0.1	***
	EC (mS cm^–1^)	0.11 ± 0.05	0.23 ± 0.08	**	2.1 ± 0.2	***
	CEC (meq 100 g^–1^)	2.3 ± 0.4	2.1 ± 0.3		3.2 ± 0.3	**

a Asterisks indicate a significance difference in the unmodified substrate compared to the augmented and amended substrates at the 0.05 (*), 0.01 (**), and <0.001 (***) levels.

### 3.4 Elemental Shifts of Filter Material

#### 3.4.1 Total Elemental Concentrations

**Figure 3:**
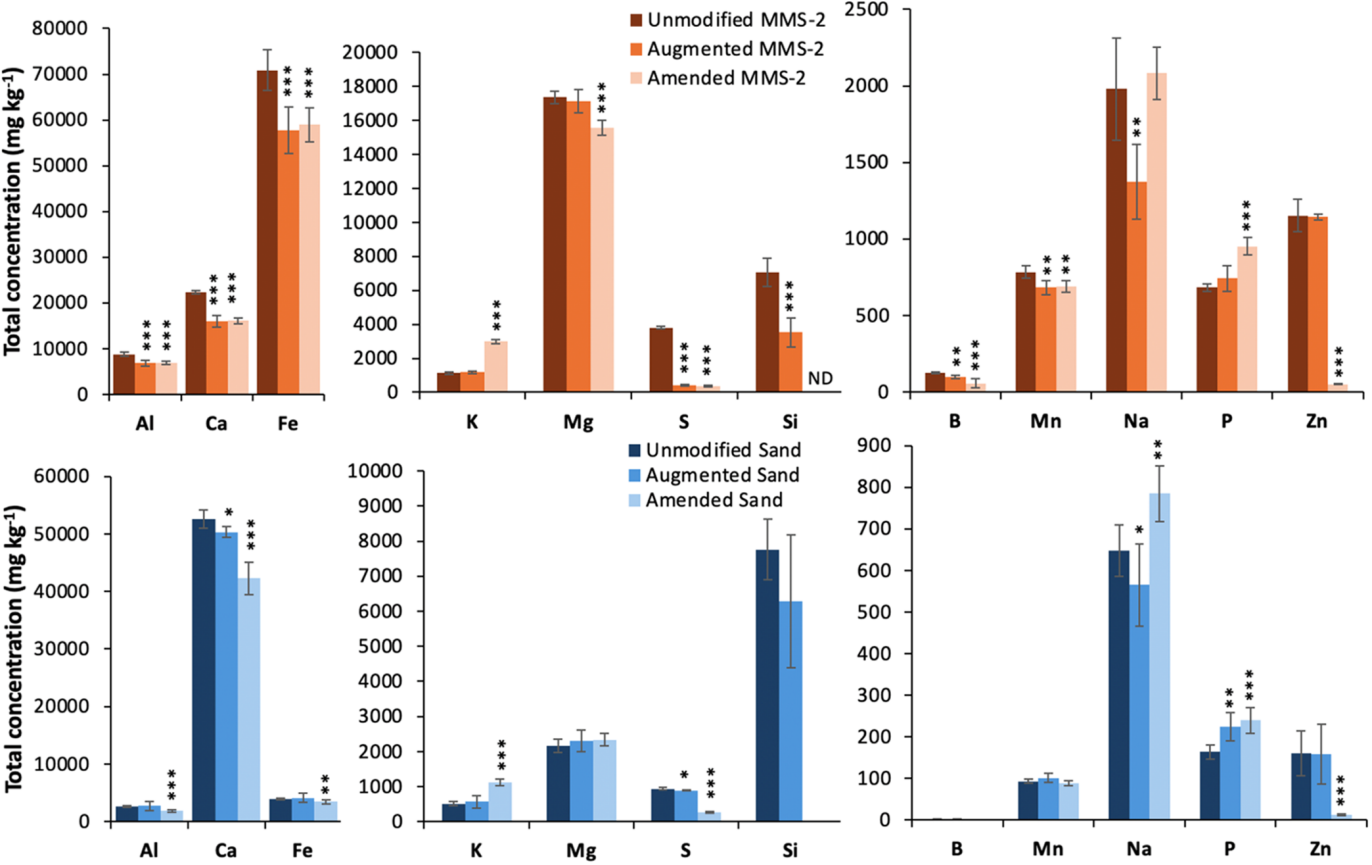
Total elemental concentrations reported as means ± standard deviation (SD) in MMS-2 and sand with an unmodified substrate, aeroponically augmented with root rinsate, or amended with residues after augmentation. Asterisks indicate a significance difference in the unmodified substrate compared to the augmented and amended substrates at the 0.05 (*), 0.01 (**), and 0.001 (***) levels.

Total elemental concentrations were altered more in MMS-2 than in sand as an effect of aeroponic augmentation. Reduction in total elemental concentrations in the aeroponically augmented treatment indicates net dissolution into solution, whereas increases indicate net sorption. Dissolution of elements during aeroponic augmentation can broadly be considered to be those elements becoming plant available during aeroponic growth, whereas sorption to regolith can broadly be considered those elements removed from nutrient solution and during aeroponic growth but later available for plant uptake during solid-substrate growth. The elements Al, B, Ca, Fe, Mn, and S were reduced in the MMS-2 augmented and amended treatments compared to the unmodified substrate. After being amended, there were increases in K, P, and Na and a decrease in Zn in MMS-2. There was also an increase in Cu in amended MMS-2 although it is not shown in the figure due to scale; there was 6 ± 2 mg kg^–1^ in unmodified and augmented substrates and 68 ± 18 mg kg^–1^ in amended. In the sand substrate, the effect of aeroponic augmentation led to decreases in Ca, Na, and S, and an increase in P compared to the unmodified substrate. After amendment, the sand substrate had decreased Al, Ca, Fe, S, and Zn, while K, P, and Na were increased compared to the unmodified substrate (Figure 3).

#### 3.4.2 Extractable Elemental Concentrations

Extractable soil elements were more similar between substrates than between total concentrations. In MMS-2, the effect of aeroponic augmentation led to decreased Ca, Fe, Mg, and S but increased K, while amended material had increased Fe, K, and P, and decreased S and Mg compared to the unmodified substrate. In aeroponically augmented sand, Ca and Fe decreased while K, Mg, Na, P, and S increased compared to unmodified substrate, and the amended treatment showed the same statistical changes except that Fe increased and Na became below detection limit (BDL) (Table 4).

**Table 4:** Soil Mehlich-III Extractable Elemental Concentrations Reported as Means ± Standard Deviation (SD) of an Unmodified Substrate, Aeroponically Augmented with Root Rinsate, or Amended with Residues after Augmentation^a^

material	element	unmodified (mg kg^–1^)	augmented (mg kg^–1^)	*P*	amended (mg kg^–1^)	*P*
MMS-2	Al	BDL	1.0 ± 0.2		96 ± 9	
	B	48 ± 20	BDL		BDL	
	Ca	2611 ± 93	787 ± 213	***	2509 ± 289	
	Cu	BDL	BDL		BDL	
	Fe	11 ± 3	2.2 ± 0.1	***	67 ± 8	***
	K	65 ± 2	235 ± 16	***	1121 ± 110	***
	Mg	2120 ± 58	1361 ± 28	***	1524 ± 187	***
	Mn	9 ± 1	BDL		BDL	
	Na	78 ± 43	128 ± 20		BDL	
	P	14 ± 13	25 ± 15		102 ± 11	**
	S	1665 ± 33	63 ± 38	***	146 ± 18	***
	Si	BDL	BDL		BDL	
	Zn	0.1 ± 0.2	BDL		BDL	
sand	Al	BDL	0.2 ± 0.1		8 ± 3	
	B	17 ± 7	BDL		BDL	
	Ca	8415 ± 709	2,959 ± 87	***	4716 ± 662	***
	Cu	BDL	BDL		BDL	
	Fe	12 ± 2	1.5 ± 0.1	***	22 ± 3	***
	K	26 ± 1	54 ± 11	***	568 ± 87	***
	Mg	111 ± 10	117 ± 3	*	136 ± 17	**
	Mn	11 ± 1	BDL		BDL	
	Na	11 ± 2	52 ± 8	***	BDL	
	P	4 ± 2	26 ± 14	**	59 ± 10	***
	S	71 ± 16	163 ± 9	***	96 ± 16	**
	Si	BDL	BDL		BDL	
	Zn	0.4 ± 0.8	BDL		BDL	

a Statistical tests (*t*-test) for augmented and amended treatments were compared to the unmodified material. Asterisks indicate a significance difference in the unmodified substrate compared to the augmented and amended substrates at the 0.05 (*), 0.01 (**), and 0.001 (***) levels.

#### 3.4.3 Dissolution of Elements Into Solution after Aeroponic Augmentation

**Figure 4:**
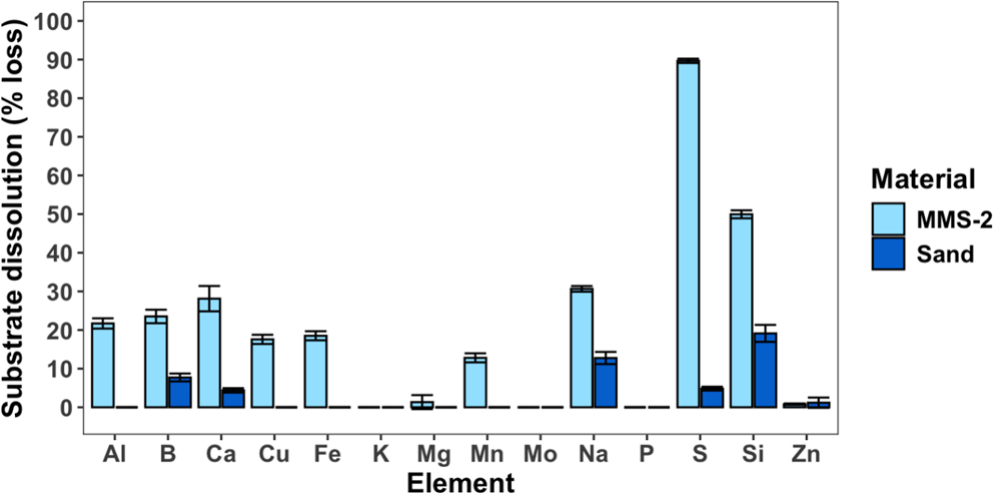
Net substrate dissolution (percent loss from original material) into aeroponic solution after aeroponic augmentation. Results are presented as the mean ± standard deviation. Elements that were accumulated (i.e., sorbed) to the regolith are displayed as having zero dissolution.

During aeroponic augmentation, the dissolution of MMS-2 and sand filter materials occurred as minerals were weathered from continuous inundation. Dissolution of filter material in the aeroponic system generates elements that are available for plant uptake. In general, MMS-2 had far greater dissolution than sand, which is also reflected by the drastic reduction of EC in augmented MMS-2. In MMS-2, the major elements dissolved into solution were S followed by Si, Na, Ca, B, Al, Fe, Cu, and Mn and in sand were Si, Na, B, and Ca (Figure 4).

#### 3.4.4 Phosphorus Speciation

**Figure 5:**
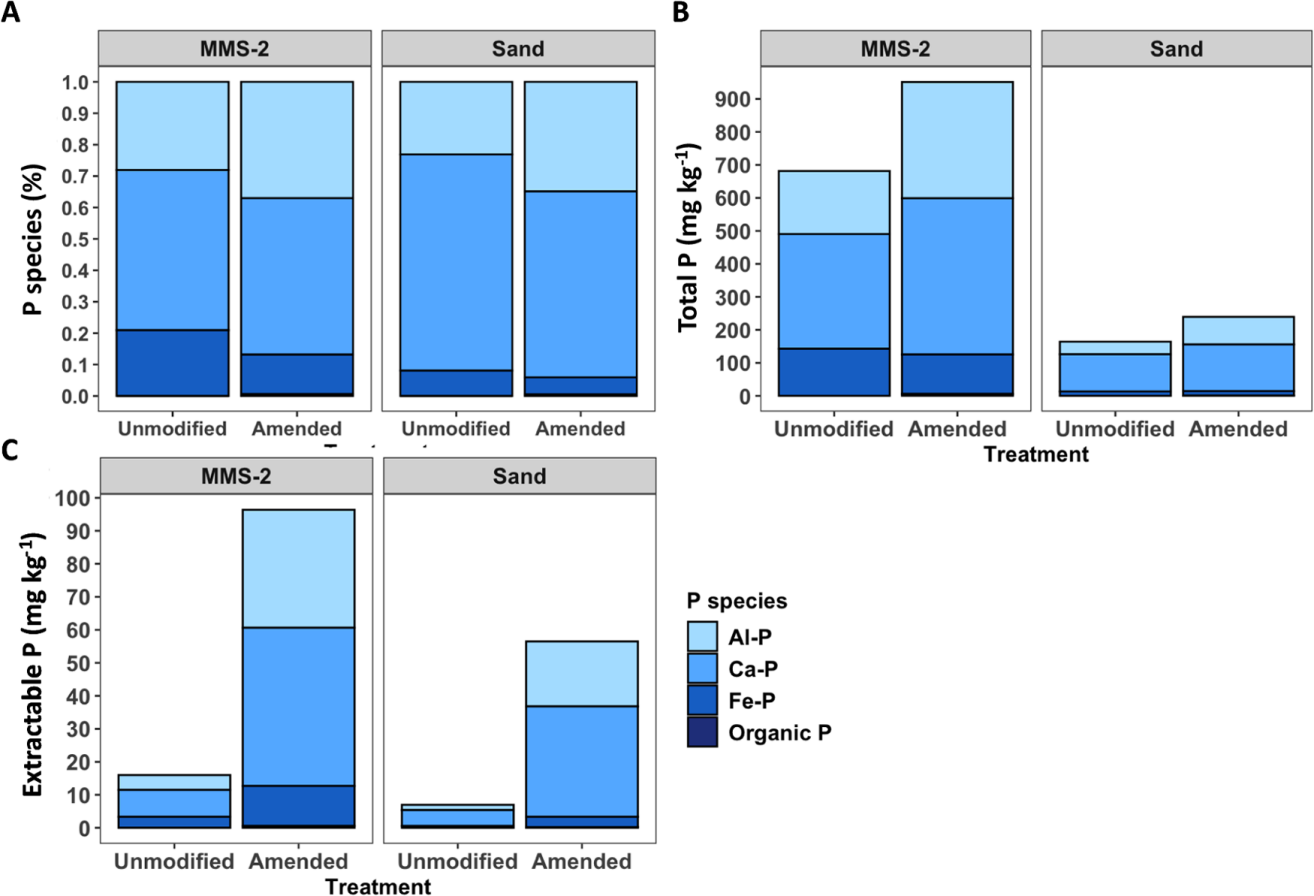
Soil P species of unmodified substrate and aeroponically augmented substrate displayed as (A) relative abundance of P species, (B) total P by P species, and (C) extractable P by P species.

Due to limited beam time, only a homogenized sample of the unmodified and amended substrates were analyzed by XANES, and no statistical analysis could be performed, thus interpretations are trends. As P increased in both MMS-2 and sand due to aeroponic augmentation and subsequent amendment with plant biomass, P speciation differed by substrate. Unfortunately, due to limited beam time, only the unmodified and amended treatments were analyzed. There was no detected organic-P (i.e., labile-P) in either unmodified substrate, but a small organic-P pool (∼1.5%) was detected in both amended substrates. In both MMS-2 and sand, changes in P species from unmodified to amended substrates were similar; Fe–P decreased slightly in amended substrates, while Ca–P remained similar (decreasing slightly in sand) and Al–P increased (Figure 5A). Coupling the P-speciation was coupled to total (Figure 5B) and extractable nutrients (Figure 5C), the trends became more apparent. It was also clear the magnitude of sorbed P was far greater in the MMS-2 substrate than sand given aeroponic augmentation and subsequent amendment, likely as a result of P in the fertilizer solution.

### 3.5 Soil Mineralogy

**Figure 6:**
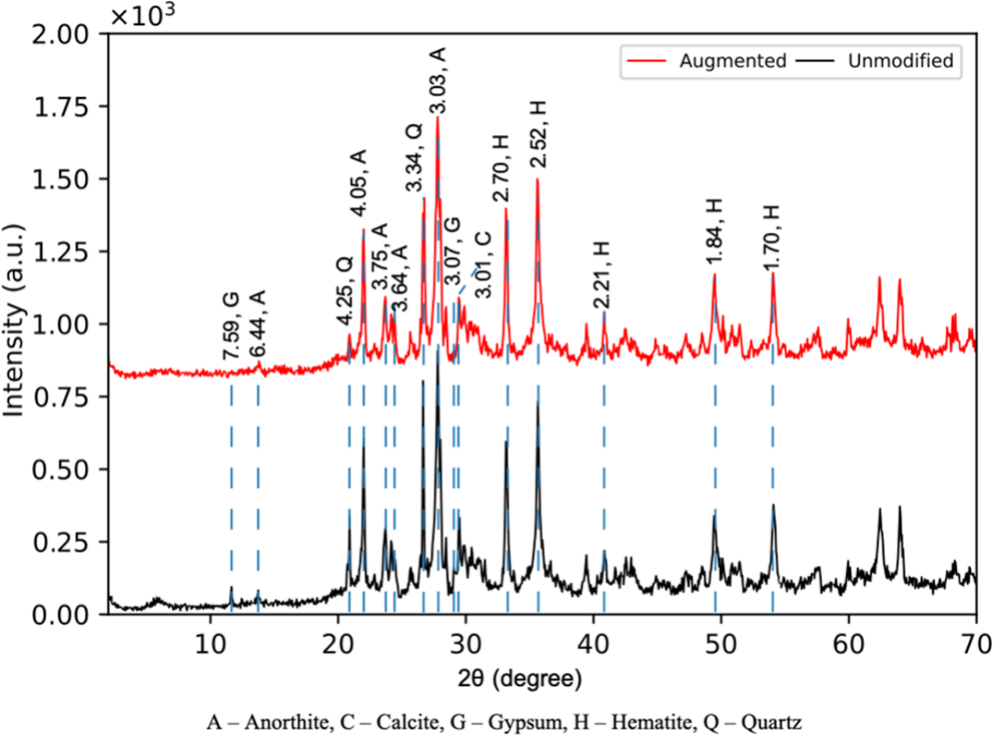
XRD patterns of unmodified (black line) and aeroponically augmented (red line) MMS-2. Peaks are labeled with *d*-spacing in angstroms. G indicates gibbsite, A indicates anorthite, Q indicates quartz, C indicates calcite, and H indicates hematite.

The mineralogy of MMS-2 in the unmodified sample includes anorthite, calcite, gypsum, hematite, and quartz, which were identified by XRD. These minerals matched well with the information provided by the supplier. After the augmented treatment, the peaks of gypsum at 7.59 and 3.07 Å disappeared (Figure 6) concluding that gypsum dissolution occurred during aeroponic augmentation. Further, the major loss of Ca and S from total elemental concentrations indicates the dissolution of gypsum. There were no mineralogical differences in the aeroponically augmented sand treatment; thus, these data are not displayed.

### 3.6 Particle Size Analysis

**Figure 7:**
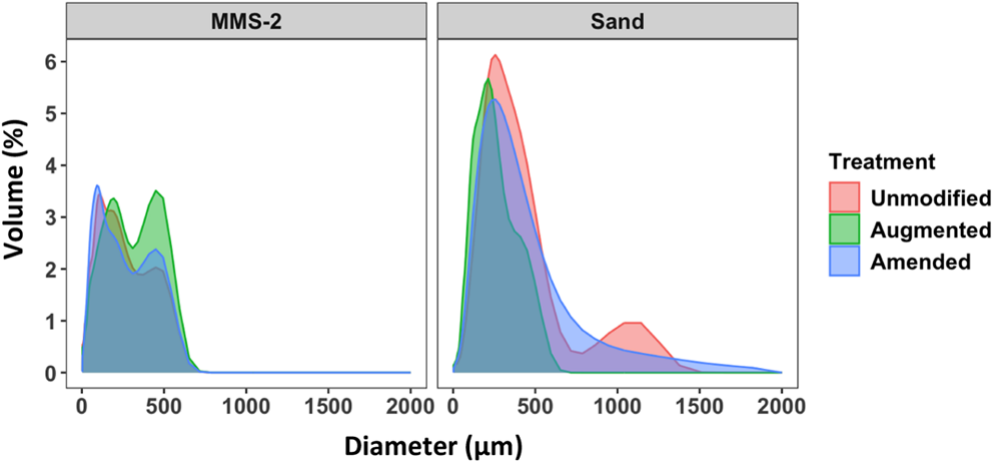
Particle size analysis of the substrates following treatments.

MMS-2 is a fine material with no fractions above 750 μm. In general, MMS-2 and sand were resistant to changes in particle size, although augmentation led to slightly increased volume around 500 μm. Unmodified sand initially had much larger grains than MMS-2 but after augmentation, there were no particles greater than 750 nm. After amendment, the sand had a greater distribution of larger particles. It is possible that small particles less than 10 μm were not captured by the analysis, nor larger aggregates created by amending the substrates with organic matter (Figure 7).

### 3.7 Growth Trial in Augmented and Amended Materials

**Figure 8:**
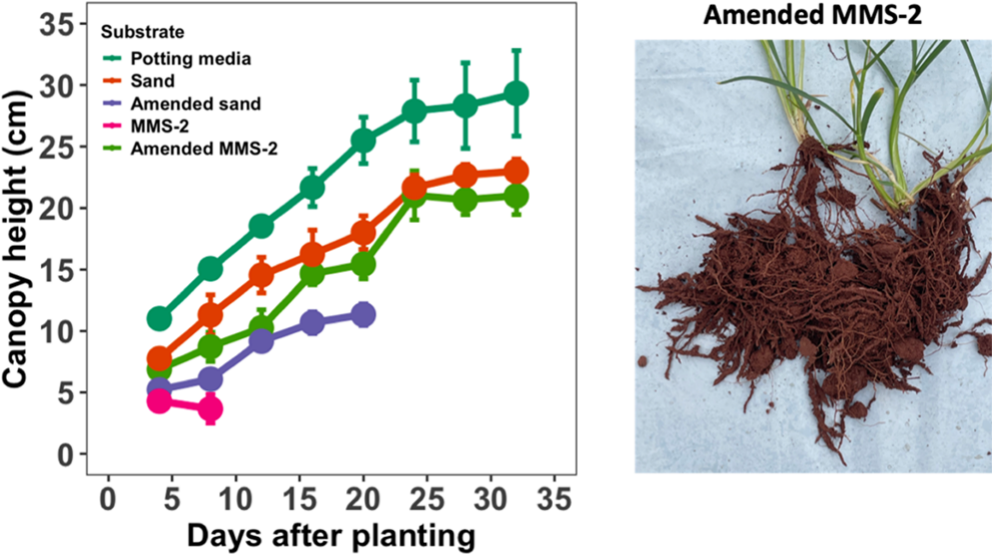
Canopy height in plants grown on different growth substrates including potting media, sand, residue-amended sand that had been augmented with root rinsate, MMS-2, and residue-amended MMS-2 that had been augmented with root rinsate. Data are presented as means ± SD. Image captured by Harrison R. Coker.

Experiments assessing plant growth in unmodified and amended substrates revealed a substrate × time interaction (*P* < 0.001). Unmodified MMS-2 was incapable of supporting plant growth as all wheat died within 10 days and was visually stressed 3–4 days after emergence. Plant growth experiments were replicated 3 times with similar wheat death in unmodified MMS-2 occurring in each trial. This is likely attributable to the lack of root system formation in the unmodified MMS-2. The amended MMS-2 treatments performed well and supported productive plant growth, which indicates that the augmentation with aeroponic biowaste and/or the residue amendment greatly improved the MMS-2 properties for plant growth. Unmodified sand led to greater plant productivity than amended sand, which eventually led to plant death after 20 days. The decrease in plant productivity in amended sand compared to unmodified sand could potentially be due to the increase in pH to 10.4, which was the highest pH of any plant growth system. Growth of higher plants in soil pH > 10 is known to impair plant productivity.^41^ High pH may also explain the improvement in plant growth from unmodified MMS-2 to amended MMS-2 as pH decreased from 10.0 to 9.6. Potting media led to the best performance among all of the substrates. Because the total substrate and pot size used for plant growth was minimal, plants were root-bound after 20 days, and growth plateaued after 24 days (Figure 8).

## 4 Discussion

In situ resource utilization of extraterrestrial regoliths will be a defining component of human colonization of planetary bodies. The described aeroponic technology and approach of utilizing regolith as a filter substrate and reincorporation of waste residues ultimately accomplished two goals for in situ resource utilization: extracting elements of interest and improving the substrate for plant growth. The rapid weathering of MMS-2 in the aeroponic system, revealed by the dissolution of gypsum and release of weatherable elements, had minimal effect on plant growth in the aeroponic system. Moreover, the amendment of residues into the augmented substrate greatly improved its ability to support plant life. Thus, the described technology succeeded in utilizing regolith as a nutrient source, evidenced by mineral dissolution, to reduce fertilizer inputs as payload from Earth; additionally, the augmented and amended Martian simulant gave rise to the growth of plants when used as a solid growth substrate whereas the unmodified material quickly led to plant death.

Dissolution of MMS-2 led to the liberation of the plant essential macronutrients Fe, Ca, S, and Mg into the recirculating aeroponic solution as well as other elements (Si, Al, and Na). It is likely that the liberated elements were biologically available. These results are synonymous with literature that weathering of lunar and Martian regolith and minerals found in regolith lead to greater abundance of plant available nutrients. In a lunar basaltic dissolution experiment, it was shown that the release of Ca, Mg, Al, Fe, and Si occurred and was increased by the addition of the organic acids citrate and oxalic acid,^4^ which are common root exudates that likely interacted with the filter media in this study. Further, higher plants grown on basalts have been shown to drastically increase the kinetics of dissolution leading to the liberation of Si, Ca, Mg, and Na, and, with some plant species, Fe.^34^ Moreover, crushed basalt has even been used as a successful fertilizer.^35^ Advanced weathering of basaltic rock has been demonstrated to supply an abundant P and K source to plants (in the absence of subsequent sorption), along with sequestration of atmospheric CO_2_;^36^ in contrast the MMS-2 used in this study showed sorption of P and K likely due to the high content of Fe/Al-oxides that are known to sorb P,^37^ which was revealed by the XANES Al–P pool increase in MMS-2. Aeroponic solution pH was maintained at 6.0–6.5 during plant growth with MMS-2 as a filter media, but the no filter control and sand filter systems required acidification. The regulation of the system pH with MMS-2 and not sand was most likely related to the greater dissolution of MMS-2 into the aeroponic system.

Although total and even extractable concentrations of elements in soils are often far different from what is taken up by plants,^38^ augmentation of MMS-2 revealed dissolution of Si, Al, and Na into solution that did not reduce plant growth. The uptake of these nonessential elements that are not included in traditional nutrient solutions may even serve beneficial aspects for the stressful environments of microgravity. Si is often accumulated by plants in similar concentrations to macronutrients (1–100 mg g^–1^ dry weight) and in monocotyledonous plants may be the primary mineral constituent.^39^ Si can also alleviate both abiotic (e.g., drought, salinity) and biotic (e.g., pests, diseases) stresses.^40^ Because Si is abundant in terrestrial soils but excluded from typical nutrient solutions, the omission of Si should be viewed as atypical of plant growth;^41^ indeed, it has been noted that an addition of 30 μM Si in hydroponics increased the edible yield, raised crop quality, and extended shelf life of corn salad (*Valerianella locusta*),^42^ and that an addition of 0.65 mM Si to hydroponics increased the vegetative growth and cuticle thickness of lettuce (*Lactuca sativa*), tomato (*Solanum lycopersicum*), sweet pepper (*Capsicum annuum*), melon (*Cucumis melo*), and cucumber (*Cucumis sativus*).^43^ Apart from Si, Na can quickly become deleterious to plant growth but when present in concentrations similar to micronutrients can promote activity of the C4 photosynthetic pathway and be a source of Na to herbivores^44^ and promotes the growth of halophytes such as beet (*Beta vulgaris*).^45^ While Al toxicity is a major concern for plants in acidic soils, there is evidence that in small concentrations Al^3+^ can induce beneficial attributes such as resistance against plant stresses and stimulation of root growth.^46^ While the elemental analysis used in this study certainly did not account for all elements likely to be present in MMS-2, sand, or an actual extraterrestrial regolith (Ti, V, Rb, Zr, rare earth elements, etc.), these may also become liberated into aeroponic solutions and nonetheless deserve further investigation for their ability to be taken up or directly reclaimed as raw feedstock. In general, the passive dissolution of MMS-2 in the aeroponic system offered a source for both essential and beneficial plant nutrients that did not hinder plant performance compared to that of the sand, which was mostly inert.

While early plant growth experiments focused primarily on the incorporation, retention, dissolution, and exchange of nutrient elements (i.e., zeolites, hydroxyapatites) to facilitate improved plant nutrition,^47−55^ more recent experiments have trended toward incorporating amendments of organic matter.^56−63^ Indeed, a recent review on plant growth in lunar and Martian simulants and methods for simulant amelioration identified that amendments of organic wastes promote beneficial properties that improve plant productivity.^57^ Corresponding to these results, aeroponically augmented and subsequently amended MMS-2 was capable of supporting wheat growth, whereas the unmodified MMS-2 led to quick plant death. At the time of analysis, there were no significant increases in C and N in the aeroponically augmented MMS-2 or sand, which may be due to rapid degradation from microbial activity. However, visually inspecting the filter material revealed small root particles that were captured, which may have aided the system from unattended biological growth in pumps and reservoirs and clogging of spray nozzles. A drawback of the current prototype was that not enough MMS-2 and sand were generated to allow for enough soil volume to grow wheat to its full life cycle. Wheat requires a large soil volume for the roots to explore and will sense becoming root-bound in small containers and subsequently prevent full plant growth.^64^ However, it was clear that aeroponically augmented and amended MMS-2 allowed for enhanced plant growth when unmodified MMS-2 almost immediately induced plant death. Improved plant performance in amended MMS-2 is likely due to the weathering of sharp mineral edges and increased total C, total N, and active C (POxC).

Plant growth in soilless systems requires constant nutrient inputs, and as plants grow, these nutrients will be taken up by the plant and converted to organic forms; thus, undesirable plant material, such as inedible parts, roots, etc., will require processing organic wastes into inorganic nutrients in order to reutilize those nutrients for downstream growth. Because soilless systems are therefore not sustainable, weathering the regolith as a nutrient source offers an ISRU approach to reduce fertilizer payloads from Earth. While the system here combines regolith weathering with plant growth, there is also the potential for separating the regolith from plants to obtain nutrients in a separate apparatus and then directly feeding the weathered solution containing nutrients to plants grown in a soilless system. A future direction to further establish the understanding of this technology would be to understand how root-associated compounds influence mineral dissolution compared to water.

The use of a pressurized aeroponic system will be practical for reduced gravity environments; thus, the current approach satisfies a working prototype that can be scaled to a higher technology readiness level (TRL). Further improvements to the system could be the diversion of aeroponic solution for direct extraction of elements of interest and identifying the degree to which nutrient solutions can be supplemented by dissolved regolith. For health concerns, perchlorate salts that exist up to 0.6 wt % on Mars were not added to the system. Although perchlorates are highly soluble and could be leached from Martian regolith prior to use in the aeroponic system, it would be interesting to explore if phytodegradation or uptake of perchlorate^65^ could occur during aeroponic plant growth. Finding microorganisms capable of biological perchlorate reduction^66^ and that could survive in niches in the aeroponic system may be another feature to add to the proposed technology. Additionally, terrestrial applications of the described aeroponic technology may be relevant to reducing fertilizer inputs, given the success of releasing nutrients from the substrate-filled filter. Because the aeroponic system described can liberate elements through rapid weathering of the regolith while simultaneously improving an unproductive regolith for plant growth, there is reason to consider and further develop such technology for use in lunar and Martian colonies.
